# Glycosylphosphatidylinositol-Anchored Proteins in *Arabidopsis* and One of Their Common Roles in Signaling Transduction

**DOI:** 10.3389/fpls.2019.01022

**Published:** 2019-08-29

**Authors:** Ke Zhou

**Affiliations:** FAFU-UCR Joint Center for Horticultural Biology and Metabolomics, Haixia Institute of Science and Technology, Fujian Agriculture and Forestry University, Fuzhou, China

**Keywords:** glycosylphosphatidylinositol (GPI), GPI-anchored protein (GPI-AP), receptor-like kinase (RLK), ligand, signaling transduction

## Abstract

Diverse proteins are found modified with glycosylphosphatidylinositol (GPI) at their carboxyl terminus in eukaryotes, which allows them to associate with membrane lipid bilayers and anchor on the external surface of the plasma membrane. GPI-anchored proteins (GPI-APs) play crucial roles in various processes, and more and more GPI-APs have been identified and studied. In this review, previous genomic and proteomic predictions of GPI-APs in *Arabidopsis* have been updated, which reveal their high abundance and complexity. From studies of individual GPI-APs in *Arabidopsis*, certain GPI-APs have been found associated with partner receptor-like kinases (RLKs), targeting RLKs to their subcellular localization and helping to recognize extracellular signaling polypeptide ligands. Interestingly, the association might also be involved in ligand selection. The analyses suggest that GPI-APs are essential and widely involved in signal transduction through association with RLKs.

## Glycosylphosphatidylinositol (GPI) Modification and GPI-Anchored Protein (GPI-AP) Biosynthesis

**Figure 1:**
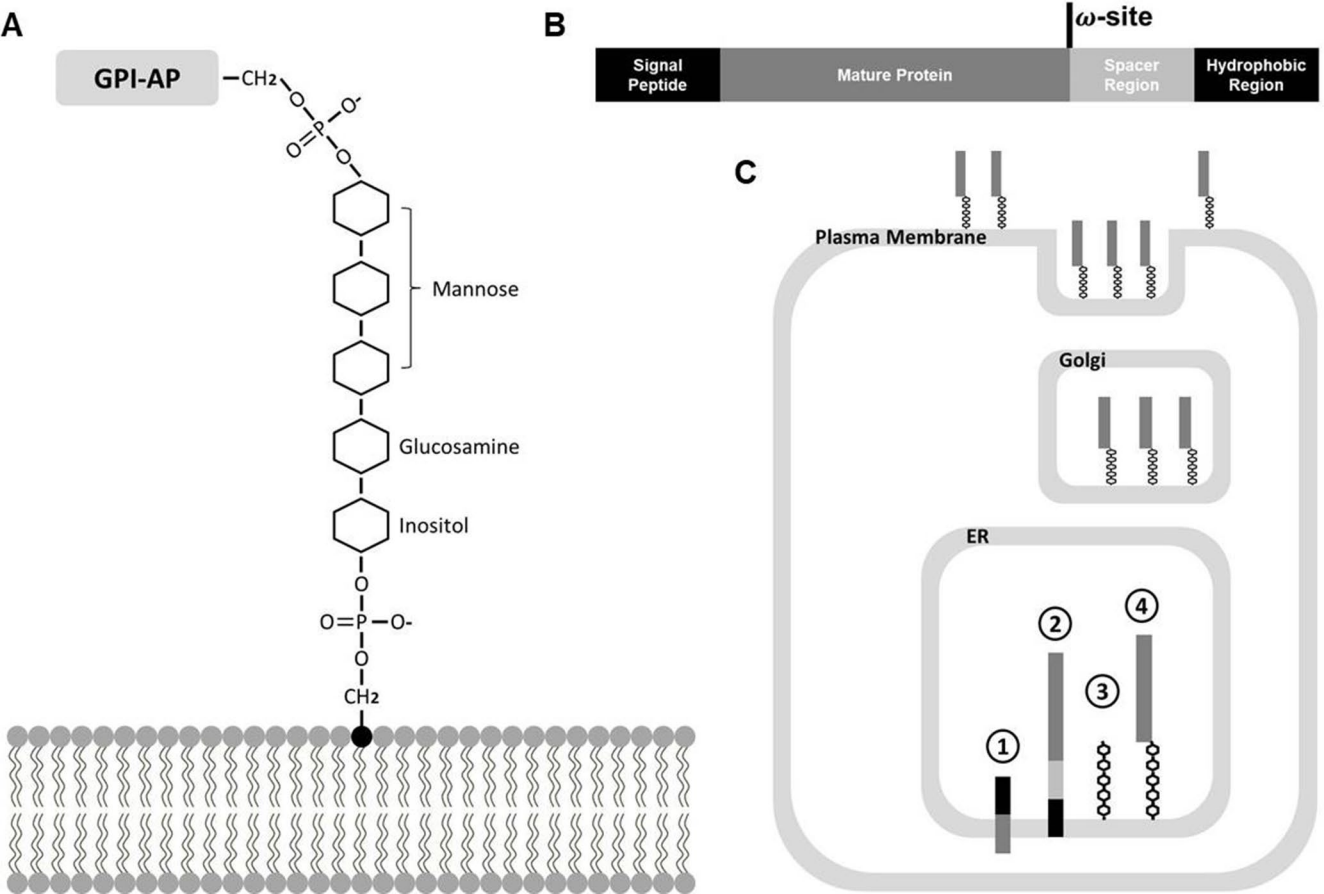
Biosynthesis of GPI moiety and GPI-AP. **(A)** Minimal oligosaccharide structure of GPI modification, D-Manα (1–2)-D-Manα (1–6)-D-Manα (1–4)-D-GlcN-inositol, which covalently links the C-terminus of GPI-AP and lipid molecule. **(B)** Common structure of GPI-AP precursor. The hydrophobic regions at the N and C termini are in black and the spacer region is in light gray. **(C)** Biosynthesis of GPI-APs. ① Precursor of GPI-AP enters ER, ② C terminus of GPI-AP precursor is recognized when entering ER, ③ Oligosaccharide structure of GPI modification is synthesized separately, ④ Recognized C terminus of GPI-AP is cleaved and covalently linked to the GPI moiety.

The GPI oligosaccharide structure is ubiquitous among eukaryotes with a common minimal backbone consisting of three mannoses, one non-N-acetylated glucosamine (GlcN), and inositol phospholipid, which covalently links the carboxyl terminus (C terminus) of GPI-APs to the lipid bilayer (**Figure 1A**) (Stevens, 1995; Oxley and Bacic, 1999; Kinoshita and Fujita, 2016). Catalyzed by a series of enzyme complexes, GPI biosynthesis starts with a lipid molecule at the rough side of the endoplasmic reticulum (ER), and this then flips and the synthesis is completed on the luminal side of the ER (**Figure 1C**) (Stevens, 1995; Takeda and Kinoshita, 1995; Kinoshita and Fujita, 2016). The typical GPI-AP precursors possess a common structure that lead them to be modified by GPI moieties inside endomembrane systems: amino-terminal (N-terminal) hydrophobic signal peptides lead them to enter the ER lumen, and during translation and maturation, C-terminal hydrophobic signals are recognized and cleaved at the ω position by a series of catalytic complexes, where the peptide bond is replaced by a bond with ethanolamine phosphate (**Figures 1A–C**) (Eisenhaber et al., 1998; Kinoshita, 2014a; Kinoshita and Fujita, 2016).

The GPI moiety allows these GPI-APs, which possess no transmembrane region, to be anchored to membrane lipid bilayers. Compared to transmembrane association, GPI anchoring has its advantages: GPI-AP shedding and release due to the presence of GPI-specific phospholipases (PLC) makes this association reversible in mammalian cells (Orihashi et al., 2012; Fujihara and Ikawa, 2016). In plants, although similar shedding and release mechanisms are indicated as various GPI-APs were identified in cell walls, thus far, no GPI-specific PLC has been identified yet (Bayer et al., 2006; Yeats et al., 2018). However, a bacterial phosphatidylinositol-specific PLC (PI-PLC) has been used for shedding GPI-APs from lipid bilayers *in vitro* and identifying them by further proteomic analysis in *Arabidopsis* (Borner et al., 2003; Elortza et al., 2003; Takahashi et al., 2016; Yeats et al., 2018).

## Importance of GPI Anchoring for GPI-APs

GPI-APs and their GPI moieties were demonstrated to be crucial for diverse developmental processes in mammals and in plants, because development was found to be broadly and severely affected if GPI moiety biosynthesis is disrupted (Kawagoe et al., 1996; Gillmor et al., 2005; Kinoshita, 2014b; Bundy et al., 2016).

As the most noticeable feature, GPI anchoring was thought to be essential for the functions of GPI-APs, and their enzymatic activities or subcellular localizations could be altered by the removal of the GPI moiety (Tozeren et al., 1992; Butikofer et al., 2001; Davies et al., 2010). However, there are exceptions: the GPI anchoring of ZERZAUST and FLA4/SOS5 was shown to be dispensable for their functions in *Arabidopsis* (Vaddepalli et al., 2017; Xue et al., 2017).

GPI moieties also play crucial roles for driving the transient, relatively ordered membrane domains rich in sphingolipids and sterols, which are called lipid rafts or microdomains, to their target regions (Saha et al., 2016; Sezgin et al., 2017; Hellwing et al., 2018; Lebreton et al., 2018). In mammalian and yeast cells, GPI-APs are co-clustered and organized in a mixture of monomers and cholesterol-dependent nanoclusters in the same lipid raft. These exit the ER in vesicles distinct from other secretory proteins and are predominantly sorted to the apical surface to serve in protein trafficking and signaling transduction (Eisenhaber et al., 1998; Morsomme et al., 2003; Legler et al., 2005; Muniz and Zurzolo, 2014; Miyagawa-Yamaguchi et al., 2015; Sezgin et al., 2017). In *Arabidopsis*, although GPI modification was found essential for protein delivery from the ER to ht eplasmodesmata (Zavaliev et al., 2016), the lipid raft mechanism has not been well revealed yet.

## Prediction and Identification of GPI-APs in *Arabidopsis*

To identify GPI-APs, various bioinformatics tools were developed, generally depending on the prediction of a specific hydrophobic region at the C terminus. Examples are big-PI Plant Predictor (http://mendel.imp.ac.at/sat/gpi/gpi_server.html) (Eisenhaber et al., 1998), PredGPI (http://gpcr2.biocomp.unibo.it/gpipe/info.htm) (Pierleoni et al., 2008), GPI-SOM (http://gpi.unibe.ch/) (Fankhauser and Maser, 2005), and fragAnchor (Poisson et al., 2007). According to the latest genomic scanning by these tools, among lower and higher eukaryotes, about 0.21% to 2.01% of total proteins from diverse families are predicted to be modified by GPI moieties, and the percentage in *Arabidopsis* is 0.83% (Eisenhaber et al., 2001; Poisson et al., 2007). In the meantime, proteomic assays, which depend on cleavage from membranes by bacterial PI-PLC treatment *in vitro* and enrichment in particular membrane fractions, were performed to compare proteomic data to bioinformatic data. To date, more than 300 GPI-APs have been identified in *Arabidopsis* (Borner et al., 2002; Borner et al., 2003; Elortza et al., 2003; Bayer et al., 2006; Takahashi et al., 2016).

*Arabidopsis* GPI-APs identified in 2003 (Borner et al., 2003; Elortza et al., 2003) and 2016 (Takahashi et al., 2016) are assembled in **Tables 1** and **2**, respectively, and their functions are discussed.

**Table 1:** A review of predicted GPI-APs updated from (Borner et al., 2003; Elortza et al., 2003).

Group	Sub-group	Total	Gene No.	Name	Descriptions
AGP	Classical AGP	17	At1g68725	AGP19	AGP17-19 encode a subclass of lysine-rich AGPs, among which AGP18 was reported to be essential for the initiation of female gametogenesis both at the sporophytic and gametophytic levels, and AGP19 functions in cell division and expansion (Acosta-Garcia and Vielle-Calzada, 2004; Sun et al., 2005; Yang et al., 2007; Yang et al., 2011; Zhang et al., 2011a; Zhang et al., 2011b).
			At4g37450	AGP18	
			At2g23130	AGP17	
			At5g14380	AGP6	AGP6 and AGP10 are co-expressed and co-localized in pollen grains and pollen tubes and essential for pollen grain development and pollen early germination, possibly because they are essential components of the nexine layer in pollen cell wall (Levitin et al., 2008; Coimbra et al., 2009; Coimbra et al., 2010; Costa et al., 2013; Palareti et al., 2016).
			At4g09030	AGP10	
			At3g01700	AGP11	Its GPI modification has been experimentally confirmed, but its function has not been characterized yet (Schultz et al., 2000).
			At5g64310	AGP1	
			At2g22470	AGP2	
			At4g40090*	AGP3	Shown as At4g40091 in Borner et al. (2003).
			At5g10430	AGP4/JAGGER	Essential for the degeneration of synergid cells, which guide the pollen tube attraction after acceptance of the unique pollen tube, and for prohibition of polytubey (Pereira et al., 2016a; Pereira et al., 2016b).
			At1g35230	AGP5	
			At5g65390	AGP7	
			At2g14890	AGP9	
			At5g18690	AGP25	
			At2g47930	AGP26	
			At3g06360	AGP27	
			At4g16980*		Shown as At4g16985 in Borner et al. (2003).
	AG peptides (Schultz et al., 2004), a group of GPI-anchored arabinogalactan polypeptides	12	At3g13520	AGP12	
			At4g26320	AGP13	
			At5g56540	AGP14	AGP14 and At3g01730 regulate root hair elongation exhibiting environmental response behavior, potentially by controlling root hair cell wall synthesis (Lin et al., 2011).
			At3g01730		
			At5g11740	AGP15	
			At2g46330	AGP16	
			At3g61640	AGP20	
			At1g55330	AGP21	
			At5g53250	AGP22	
			At3g57690	AGP23	
			At5g40730	AGP24	
			At3g20865	AGP40	
	FLAs (fasciclin-like AGPs)	16	At5g55730	FLA1	Involved in lateral root initiation and shoot regeneration potentially by regulating cell-type specification (Johnson et al., 2011).
			At4g12730	FLA2	
			At2g24450	FLA3	Specifically expressed in pollen grains and tubes and involved in microspore development potentially through the regulation of cellulose deposition (Li et al., 2010).
			At3g46550	FLA4/SOS5	Directly associates with cell wall RLKs FEI1/2 to perceive environmental stimuli in apoplast by altering its conformation and association with FEI1/2. This complex could regulate cell wall synthesis and composition by collaborating with CESA5. Interestingly, this regulation could also be controlled by ethylene and ABA with unclear mechanism. Surprisingly, the absence of GPI anchors only affected their PM localization but not their function (Harpaz-Saad et al., 2012; Seifert et al., 2014; Basu et al., 2016; Griffiths et al., 2016; Xue et al., 2017; Turupcu et al., 2018).
			At4g31370	FLA5	
			At2g20520	FLA6	
			At2g04780	FLA7	
			At2g45470	FLA8/AGP8	
			At1g03870	FLA9	Response to drought stress in maize and *Arabidopsis* (Cagnola et al., 2018).
			At3g60900	FLA10	
			At5g03170	FLA11	FLA11 and FLA12 affect cellulose deposition and formation of secondary cell wall composition (Ito et al., 2005; MacMillan et al., 2010).
			At5g60490	FLA12	
			At5g44130	FLA13	
			At3g12660	FLA14	
			At1g30800		
			At4g12950		
Extensin related	Extensin related	7	At1g02405		Proline-rich protein Proline-rich protein Proline-rich protein Proline-rich protein
			At1g70990		
			At4g16140		
			At5g11990		
			At3g06750		Hydroxyproline-rich glycoprotein family protein Hydroxyproline-rich glycoprotein family protein
			At1g23040		
			At5g49280		Hydroxyproline-rich glycoprotein family protein
Phytocyanins (Nersissian et al., 1998)	Stellacyanin like (Hart et al., 1996)	4	At5g20230	BCB/SAG14	Regulates lignin biosynthesis induced by oxidative stress (Ezaki et al., 2005; Kim et al., 2011; Ji et al., 2015; Tang et al., 2016).
			At2g31050		Copredoxin superfamily protein Copredoxin superfamily protein
			At2g26720		
			At5g26330		Copredoxin superfamily protein
	Uclacyanin like	8	At1g22480		Copredoxin superfamily protein
			At1g72230		Copredoxin superfamily protein
			At3g27200		Copredoxin superfamily protein
			At2g32300	UCC1	UCC1, UCC2 and UCC3 encode copper binding proteins (Nersissian et al., 1998).
			At2g44790	UCC2	
			At3g60280	UCC3	
			At3g60270		Copredoxin superfamily protein
			At5g07475		Copredoxin superfamily protein
	ENODL (early nodulin like)	17	At5g53870	ENODL1	
			At4g27520	ENODL2	Catalyzes the formation of pyroglutamic acid at the N-terminus of several peptides and proteins (Schilling et al., 2007).
			At4g28365	ENODL3	
			At4g32490	ENODL4	
			At3g18590	ENODL5	
			At1g48940	ENODL6	
			At1g79800	ENODL7	
			At1g64640*	ENODL8	Classified as unknown/hypothetical in Borner et al. (2003).
			At3g20570	ENODL9	Involved in starch mobilization and reproductive progresses (Khan et al., 2007).
			At2g23990	ENODL11	
			At4g30590	ENODL12	
			At5g25090	ENODL13	
			At2g25060	ENODL14	ENODL14 and ENODL15 directly interact with RLK FERONIA and regulate maternally controlled male-female communication and fertilization (Escobar-Restrepo et al., 2007; Hou et al., 2016).
			At4g31840	ENODL15	
			At3g01070	ENODL16	
			At5g15350	ENODL17	
			At1g08500	ENODL18	
COBRA family	COBRA family [all 12 COBRA members, except COBL5, was predicted to be GPI-AP (Roudier et al., 2002)]	10	At5g60920	COBRA/COB	Localizes on plasma membrane polarly and regulates cell wall biosynthesis and cellulose microfibrils in *Arabidopsis* and tomato (Schindelman et al., 2001; Roudier et al., 2005). Its regulation responses to various stresses potentially by involving in jasmonic acid-related signaling pathway (Ko et al., 2006; Dinneny et al., 2008; Sorek et al., 2015).
			At3g02210	COBL1	
			At3g29810	COBL2	Plays a role in the deposition of crystalline cellulose in secondary cell wall structures during seed coat epidermal cell differentiation, and the regulation is independent of the FEI-SOS pathway (Ben-Tov et al., 2015; Ben-Tov et al., 2018).
			At5g15630	COBL4/IRX6	Participates in regulating secondary cell wall biosynthesis (Taylor-Teeples et al., 2015; Niu et al., 2018).
			At1g09790	COBL6	
			At3g16860	COBL8	
			At3g20580	COBL10	Crucial for pollen tube directional growth by affecting the deposition of the apical pectin cap and cellulose microfibrils of pollen tubes and might also be involved in male-female communications (Li et al., 2013).
			At4g16120	COBL7/SEB1	
			At4g27110	COBL11	
			At5g49270	COBL9/DER9/SHV2	Involved in ethylene and auxin controlled root hair development (Parker et al., 2000; Ringli et al., 2005; Jones et al., 2006).
GPDL glycerophosphodiester phosphodiesterase like (GDPD-like) family (Cheng et al., 2011)		6	At1g66970	GDPDL1/SHV3-Like2/SVL2	Homologue of the extracellular domain of RLK GDPDL2/AT1g66980. Possesses the capacity to hydrolyze glycerophosphodiesters, which is stimulated by Ca^2+^ in *Arabidopsis*, and plays an important role in various physiological processes (Cheng et al., 2011).
			At3g20520	GDPDL5	
			At4g26690	GDPDL3/SHV3	SHV3 and GDPDL4 are involved in primary cell wall organization, which regulates cell polar expansion by coordinating proton pumping and cellulose synthesis (Parker et al., 2000; Hayashi et al., 2008; Yeats and Somerville, 2016; Yeats et al., 2016).
			At5g55480	GDPDL4/SVL1	
			At5g58050	GDPDL6/SVL4	
			At5g58170	GDPDL7/SVL5	
HIPL		3	At1g74790		
			At5g39970		
			At5g62630	HIPL2	
β-1,3 Glucanas-es		31	At1g11830**	Does not exist	Shown in Borner et al. (2003) but could not be found in databases
			At1g26450		Carbohydrate-binding X8 domain superfamily protein
			At1g64760	ZERZAUST, ZET	Required for cell wall organization during tissue morphogenesis potentially by being mediated by RLKs. Interestingly, the presence of GPI anchor is dispensable for its function (Fulton et al., 2009; Vaddepalli et al., 2017; Vaddepalli et al., 2019).
			At2g19440	ZETH	Homolog of ZET and works redundantly with ZET (Vaddepalli et al., 2019).
			At1g32860		Glycosyl hydrolase superfamily protein
			At1g77780		Glycosyl hydrolase superfamily protein
			At2g26600		Glycosyl hydrolase superfamily protein
			At3g15800		Glycosyl hydrolase superfamily protein
			At1g66250		O-Glycosyl hydrolase family 17 protein
			At2g01630		O-Glycosyl hydrolase family 17 protein
			At3g04010		O-Glycosyl hydrolase family 17 protein
			At3g13560		O-Glycosyl hydrolase family 17 protein
			At3g24330		O-Glycosyl hydrolase family 17 protein
			At4g29360		O-Glycosyl hydrolase family 17 protein
			At4g31140		O-Glycosyl hydrolase family 17 protein
			At5g18220		O-Glycosyl hydrolase family 17 protein
			At5g20870		O-Glycosyl hydrolase family 17 protein
			At5g42720		O-Glycosyl hydrolase family 17 protein
			At5g56590		O-Glycosyl hydrolase family 17 protein
			At5g58090		O-Glycosyl hydrolase family 17 protein
			At5g58480		O-Glycosyl hydrolase family 17 protein
			At5g64790		O-Glycosyl hydrolase family 17 protein
			At5g42100	BG_PPAP	Regulates the gating of plasmodesmata and the palsmodesmatal transport through plasmodesmal callose degradation (Zavaliev et al., 2016).
			At5g61130	PDCB1	PCDB1-PDCB3, At1g69295, and At3g58100 encode a subgroup of X8-domian containing GPI-APs, which localize to the plasmodesmata and predicted to bind callose (Simpson et al., 2009; Zavaliev et al., 2016).
			At5g08000	PDCB2	
			At1g18650	PDCB3	
			At1g69295		
			At3g58100		
			At1g09460***		At1g09460, At2g30933, At2g03505, and At4g13600 encode a subgroup of X8-domain-containing GPI-APs (Simpson et al., 2009) but not included in Borner et al. (2003).
			At2g30933***		
			At2g03505***		
			At4g13600***		
Polygalacturonase		1	At3g15720		Pectin lyase-like superfamily protein
Pectate lyases		3	At3g53190		Pectin lyase-like superfamily protein
			At3g54920	PMR6	Required for fungal infection progress and effects cell wall composition through pectin synthesis (Vogel et al., 2002; Vogel et al., 2004).
			At5g04310		Pectin lyase-like superfamily protein
Proteases	Aspartyl proteases	10	At1g05840		
			At1g08210		
			At5g36260	A36	A36 and A39 co-localize with GPI-anchored COBL10 and involved in pollen tube germination, vitality, and pollen tube guidance (Gao et al., 2017; Gao et al., 2017).
			At1g65240	A39	
			At2g17760		
			At3g02740		
			At3g51330		
			At3g51350		
			At4g35880		
			At5g10080		
	Metalloproteases	5	At1g24140	AT3-MMP	This subgroup of proteases contribute to the MAMP-triggered callose deposition in response to the bacterial flagellin peptide flg22, which suggests their involvement in the pattern-triggered immunity in interactions with necrotrophic and biotrophic pathogen (Zhao et al., 2017).
			At1g59970	AT5-MMP	
			At1g70170	AT2-MMP	
			At2g45040	AT4-MMP	
			At4g16640	AT1-MMP	
	Cys proteases	1	At3g43960		Regulates root hair elongation (Lin et al., 2011).
LTPL (lipid transfer-like protein)		26	At1g05450		
			At1g18280	LTPG3	
			At1g27950	LTPG1/LTPG	LTPG1, LTPG2, LTPG5, and LPTG6 are involved in cuticular wax export or accumulation in epidermal cells and during pathogen defense (Debono et al., 2009; Kim et al., 2012; Guo et al., 2013; Edstam and Edqvist, 2014; Fahlberg et al., 2019).
			At3g43720	LTPG2	
			At3g22600	LTPG5	
			At1g55260	LTPG6	
			At1g62790		
			At1g73890		
			At2g13830		
			At2g27130		
			At2g44290		
			At2g44300		
			At2g48130	LTPG15	Involved in suberin monomer export in seed coats (Lee and Suh, 2018).
			At2g48140	EDA4	
			At1g36150		
			At3g22611**	Does not exist	Shown in Borner et al. (2003) but could not be found in databases.
			At3g58550		
			At4g08670	LTPG4	
			At4g12360		
			At4g14805		
			At4g14815		
			At4g22630		
			At4g22640		
			At5g09370		
			At5g13900		
			At5g64080	XYP1	XYP1 and XYP2 function as mediators of inductive cell-cell interaction in vascular development (Motose et al., 2004). XYP2 was not shown in (Borner et al., 2003) due to its alternative splicing.
			At2g13820***	XYP2	
SKU5-Similar family		4	At4g12420	SKU5	SKU5 is involved in root directional growth (Sedbrook et al., 2002), and this group of genes is redundantly essential for root development by regulating cell polar expansion and cell wall synthesis (Zhou, 2019a, Zhou, 2019b). SKS3 was not shown in Borner et al. (2003) due to alternative splicing.
			At4g25240	SKS1	
			At5g51480	SKS2	
			At5g48450***	SKS3	
RLP	RLK3 like (DUF26)	5	At1g63550		This subgroup of RLPs homolog with the extracellular region of a group of cysteine-rich RLKs (CRKs).
			At1g63580		
			At5g41280		
			At5g41290		
			At5g41300		
	PRK5 like	3	At1g20030		This subgroup of pathogenesis-related thaumatin superfamily proteins are similar with the extracellular region of an osmotin/thaumatin-like protein kinase PR5K (PR5-like receptor kinase) (Wang et al., 1996; Abdin et al., 2011).
			At4g36010		
			At4g38660		
	Lectin like	1	At1g07460		Homologue of L-type lectin receptor kinase III, 1 (LECRK-III, 1)
	LysM domains	3	At1g21880	LYM1/LYP2	LYM1 and LYM3 physically interact with the major components of bacterial cell walls and peptidoglycans and work together with a LysM RLK CERK1 to mediate perception and immunity to infection (Willmann et al., 2011).
			At1g77630	LYM3/LYP3	
			At2g17120	LYM2/LYP1	
	Cf-2/Cf-5 like	3	At1g80080	ATRLP17/TMM	Forms various complexes with different transmembrane RLKs from ERECTA family (ERf) and/or SERKs to recognize their ligands, such as epidermal patterning factors (EPFs) and CHAL, and then to regulate stomatal development and immune response through the activation of intracellular MAPK cascade (Bundy et al., 2016; Abrash and Bergmann, 2010; Geisler et al., 2000; Geisler et al., 1998; Jakoby et al., 2006; Jorda et al., 2016; Kobe and Kajava, 2001; Lee et al., 2015; Lee et al., 2012; Lin et al., 2017; Meng et al., 2015; Rasmussen et al., 2011; Wang et al., 2008; Yan et al., 2014; Bhave et al., 2009).
			At2g42800	ATRLP29	
			At4g28560	RIC7	Interacts with a component of the vesicle trafficking machinery and acts as its linker with ROP2 (Jeon et al., 2008; Xu et al., 2010; Hwang et al., 2011; Hong et al., 2016). However, the presence of its GPI anchoring is doubted (Jeon et al., 2008; Yeats et al., 2018).
	Other	1	At1g10375**	Does not exist	Shown in Borner et al. (2003) but could not be found in databases.
			At4g23180***		Encoded by an alternative variant of *CRK10*, which was believed to encode a cysteine-rich RLK (Grojean and Downes, 2010). Not shown in Borner et al. (2003) due to alternative splicing.
GPI-anchored peptides	GPI-anchored peptides	8	At3g01940		
			At3g01950		
			At5g14110		
			At5g40960		
			At5g40970		
			At5g40980	AT.I.24-6	
			At5g50660		
			At5g63500		
LORELEI-like family		4	AT4g26466***	LORELEI	LLG1 chaperones transmembrane RLK FERONIA from the ER to the plasma membrane, where both LORELEI and LLG1 could associate with FERONIA to recognize extracellular ligands to regulate sperm cell release during double fertilization and early seed development (Capron et al., 2008; Duan et al., 2010; Tsukamoto et al., 2010; Meng et al., 2012; Yu et al., 2012; Li C et al., 2015; Li et al., 2016; Liao et al., 2017; Stegmann et al., 2017; Feng et al., 2018; Guo et al., 2018; Yin et al., 2018). Interestingly, LLG1 was also reported to associate with RLK FLS2 and medicate PAMP recognition (Shen et al., 2017). LORELEI was not shown in Borner et al. (2003).
			At2g20700	LLG2	
			At4g28280	LLG3	
			At5g56170	LLG1	
PLC-like phosphodiesterases		1	At5g67130*		Regulates gametophytic self-incompatibility (Qu et al., 2017). Shown as At5g67131 in Borner et al. (2003).
Other		6	At5g07190	SEED GENE 3	
			At5g62200	ATS3B	
			At5g62210	ATS3	
			At3g07390		
			At1g24520	BCP1	Active in both diploid tapetum and haploid microspores and required for pollen fertility (Theerakulpisut et al., 1991; Xu et al., 1995; Luo et al., 2000).
			At4g15460		A glycine-rich protein
Unknown/hypothetical		33	At1g54860		Identified in oil bodies purified from *Arabidopsis* seeds (Jolivet et al., 2004).
			At3g06035		
			At5g19230		
			At5g19250		
			At1g07135		A glycine-rich protein
			At1g09175		
			At3g04640		A glycine-rich protein
			At3g55790		
			At1g29980		
			At2g34510		
			At5g14150		
			At3g18050		
			At4g28100		
			At3g27410		
			At5g40620		
			At1g23050		
			At1g70985		Hydroxyproline-rich glycoprotein family protein
			At5g26290	RAMCAP	Involved in both male and female gametophytic development (Singh et al., 2017).
			At5g26300		TRAF-like protein
			At3g24518**		Natural antisense transcript overlaps with AT3G24520
			At5g35890		β-galactosidase-related protein
			At1g21090		Cupredoxin superfamily protein
			At1g56320		
			At1g61900		
			At2g28410		
			At2g29660		Zinc finger (C2H2-type) family protein
			At3g26110		Anther-specific protein agp1-like protein
			At3g44100		MD-2-related lipid recognition domain-containing protein
			At3g58890		RNI-like superfamily protein
			At3g61980	KPI-1	Putative Kazal-type serine proteinase inhibitor, which is supposed to limit and control the spread of serine proteinase activity, and function during defense mechanism (Pariani et al., 2016).
			At4g14746		Neurogenic locus notch-like protein
			At4g28085		
			At4g38140		RING/U-box superfamily protein
			At5g08210**	MIR834A	Encoded a microRNA of unknown function
			At5g14190**	Does not exist	Shown in Borner et al. (2003) but could not be found in genomic or proteomic database actually
			At5g16670**	Does not exist	Shown in Borner et al. (2003) but could not be found in genomic or proteomic database actually
			At5g22430		Pollen Olee 1 allergen and extensin family protein

*Shown incorrectly in Borner et al. (2003).

**Shown in Borner et al. (2003) but does not exist in genomic or proteomic database or encodes non-coding RNA.

***Not shown in Borner et al. (2003) but could be predicted or studied as GPI-APs.

**Table 2:** GPI-APs identified in 2016 that not included in previous study in 2003.

Group	Sub-group	Total	Gene No.	Name	Descriptions
LTPL (lipid transfer protein)		3	AT3g22620		
			AT2g13820	XYP2	Functions as a mediator of inductive cell-cell interaction in vascular development (Motose et al., 2004).
			AT4g22505		Bifunctional inhibitor/lipid-transfer protein/seed storage 2S albumin superfamily protein
β-1,3 Glucanases		3	AT1g11820		O-Glycosyl hydrolase family 17 protein
			AT4g34480		O-Glycosyl hydrolase family 17 protein
			AT3g57240		
PLC-like phosphodiesterases		1	AT4g36945		
RLP		2	AT3g17350		Wall-associated kinase family protein
			AT4g18760	RLP51/SNC2	Functions upstream of ankyrin-repeat transmembrane protein BDA1 to regulate plant immunity through transcriptional factor WRKY70 (Zhang et al., 2010; Yang et al., 2012).
Oligogalacturonide oxidase		2	AT5g66680	DGL1	Subunit of the oligosaccharyltransferase complex, which catalyzes *N*-glycosylation of nascent secretory polypeptides in the lumen of the ER (Lerouxel et al., 2005; Qin et al., 2013; Jeong et al., 2018).
			AT4g20830	ATBBE20/OGOX1	Required in plant immunity (Benedetti et al., 2018).
β-Glucuronidase		2	AT5g07830	GUS2	Contributes to the glycosylation of AGPs (Eudes et al., 2008).
			AT5g34940	GUS3	
LRR extensin		1	AT4g18670	LRX5	Physically associates with RALF peptides RALF22/23, which activate FERONIA and transduce extracellular signals to regulate plant growth and salt stress tolerance (Zhao et al., 2018).
AGP		1	AT1g28290	AGP31	Involved in cell wall structure and network (Hijazi et al., 2014).
Expansin		1	AT1g69530	EXPA1	Regulates stomatal opening by altering the structure of the guard cell wall (Wei et al., 2011; Zhang et al., 2011c).
PME and PMEI proteins	PME (pectin methylesterase)	1	AT3g14310	PME3	Catalyzes the demethylesterification of pectin homogalacturonan domains in plant cell walls, and its activity could be regulated by PMEIs (Guenin et al., 2011; Senechal et al., 2015).
	PMEI (pectin methylesterase inhibitors)	5	AT2g31430	PMEI5	A pectin methylesterase inhibitor (Muller et al., 2013).
			AT5g62360	PMEI13	Regulates root growth under cold and salt stresses (Chen et al., 2018).
			AT3g62820		Plant invertase/PMEI Inhibitor superfamily protein
			AT3g17130		Plant invertase/PMEI inhibitor superfamily protein
			AT5g62350		Plant invertase/PMEI inhibitor superfamily protein
GDSL motif esterase/acyltransferase/lipase		4	AT4g30140	CDEF1	Possesses esterase activity and candidates for the unidentified plant cutinase for cutile biosynthesis (Takahashi et al., 2010).
			AT5g45950		GDSL-motif esterase/acyltransferase/lipase
			AT4g01130		GDSL-motif esterase/acyltransferase/lipase
			AT3g16370		GDSL-motif esterase/acyltransferase/lipase
Proteases		3	AT1g30600		Subtilisin-like serine protease
			AT4g21650	SBT3.13	Subtilisin-like serine protease
			AT3g61820		Eukaryotic aspartyl protease family protein
Others		25	AT2g30700		
			AT1g65870		Disease resistance responsive
			AT5g42370		Calcineurin-like metallophosphoesterase superfamily protein
			AT2g03530	UPS2	Mediates high-affinity uracil and 5-fluorouracil (a toxic uracil analogue) transport (Schmidt et al., 2004).
			AT1g72610	GLP1	Localizes to the extracellular matrix and being considered to be involved in many physiological responses including environmental stress (Membre et al., 2000).
			AT5g19240		Glycoprotein membrane precursor GPI anchored
			AT5g04885	BGLC3	Possesses β-glucosidases activity and works redundantly with its homolog BGLC1 with absent GPI anchor to remove unsubstituted Glc residues from the nonreducing end of xyloglucan molecule (Sampedro et al., 2017).
			AT3g05910	PAE12	Pectin acetyesterase 12
			AT3g47800		Galactose mutarotase-like superfamily protein
			AT5g55750		Hydroxyproline-rich glycoprotein family protein
			AT3g53010		Carbohydrate esterase
			AT4g29520	SES1	ER localized molecular chaperone and required for heat tolerance (Guan et al., 2019).
			AT3g07570	Cytochrome B561	
			AT1g75680	GH9B7	Glycosyl hydrolase
			AT5g14030		Translocon-associated protein beta unit (TRAPB)
			AT3g29360	UGD2	Products UDP-glucuronic acid, which is the common precursor for arabinose, xylose, galacturonic acid, and apiose residues in cell wall biosynthesis (Reboul et al., 2011; Siddique et al., 2012).
			AT5g64620	C/VIF2	Inhabits both plant cell wall invertase and vacuolar invertase (Link et al., 2004).
			AT1g68560	XYL1	Releases xylosyl residues from xyloglucan oligosaccharides at the non-reducing end, which alters xyloglucan composition and results in growth defects (Sampedro et al., 2010; Gunl and Pauly, 2011).
			AT4g34180	CYCLASE1	A negative regulator of cell death and regulates pathogen-induced symptom development in *Arabidopsis* (Smith et al., 2015).
			AT4g35220	CYCLASE2	
			AT5g08260	SCPL35	Serine carboxypeptidase-like 35
			AT2g33530	SCPL46	
			AT5g43600	AAH-2/UAH	Its allantoate amidohydrolases enzymatic activity is required for nitrogen recycling from purine ring in plants (Werner et al., 2008).
			AT4g15630	CASPL1E1	
			AT4g15620	CASPL1E2	
Not typical GPI-Aps					
Transmembrane protein with predicted omega domain at C terminus		8	AT4g02420		
			AT1g53210		
			AT1g55910	ZIP11	
			AT3g48200		
			AT1g42470	ATNPC1-1	
			AT1g70940	PIN3	Auxin efflux carrier family protein
			AT2g01420	PIN4	Auxin efflux carrier family protein
			AT5g55960		
Predicted cytosol protein without signal peptide at N terminus		13	AT1g65820		Microsomal glutathione S-transferase
			AT2g45140	PVA12	
			AT2g32240	PICC	
			AT5g22780	AP-2	Adaptin family protein
			AT4g11380		
			AT5g22770	AP2A1	
			AT3g27570 AT1g06530	PMD2	Sucrase/ferredoxin-like family protein
			AT1g22882	SUN3	
			AT4g32150 AT5g11150	VAMP711 VAMP713	VAMP711 and VAMP713 are SNARE family proteins that regulate endomembrane trafficking (Leshem et al., 2010; Ichikawa et al., 2015; Xue et al., 2018).
			AT1g16240	SYP51	A SNARE family protein that interacts with aquaporin and regulates post-Golgi traffic and vacuolar sorting (De Benedictis et al., 2013; Barozzi et al., 2018).
			AT5g16830	PEP12/SYP21	A SNARE family protein involved in protein sorting and plant development (Foresti et al., 2006; Silady et al., 2008; Shirakawa et al., 2010; Touihri et al., 2011).
			AT5g46860	SGR3/SYP22	A SNARE family protein
Transmembrane RLKs		3	AT5g48380	BIR1	Functions as a negative regulator of basal immunity and cell death in *Arabidopsis* (Liu et al., 2016).
			ATIg51940	LYK3	Regulates the cross-talk between immunity and abscisic acid responses (Paparella et al., 2014).
			AT1g63430		Transmembrane RLK

**Figure 2:**
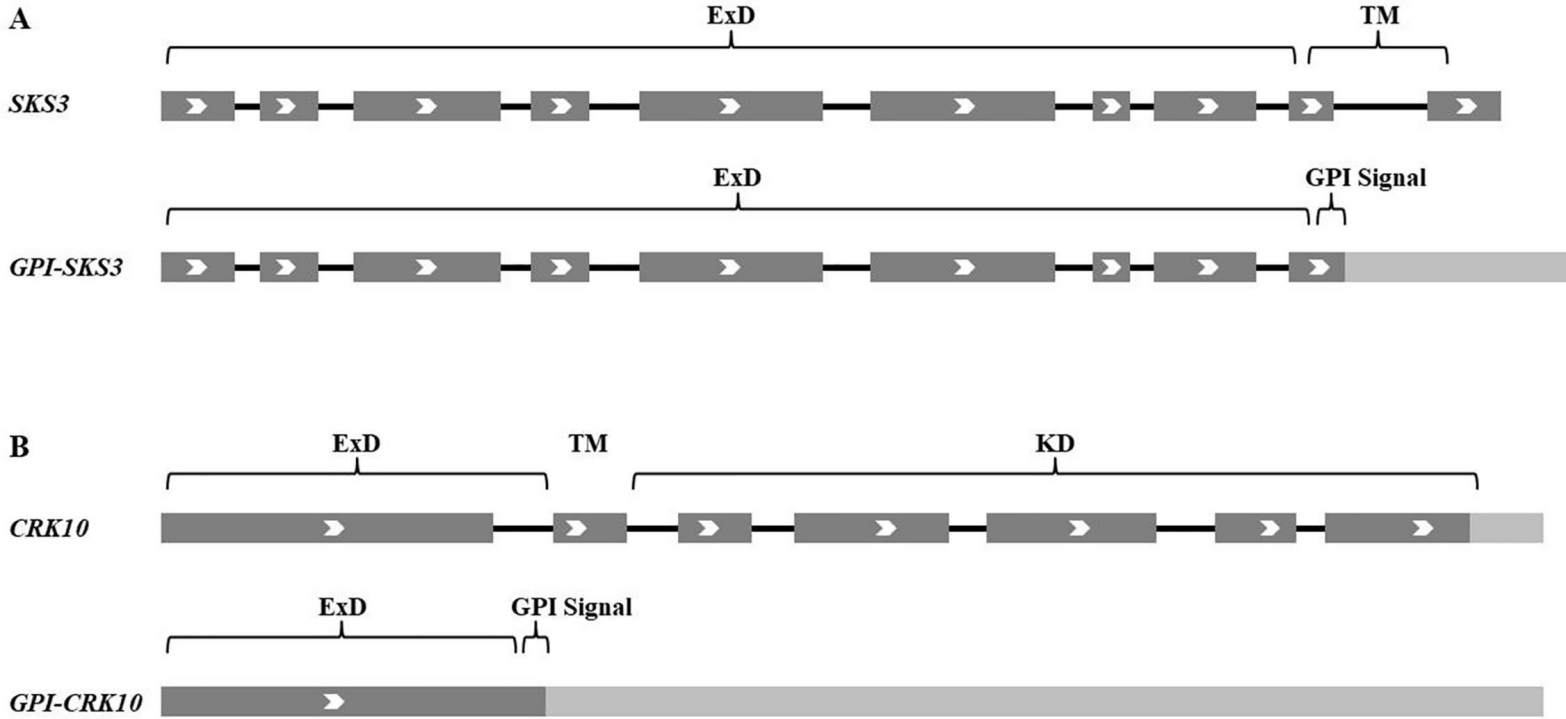
GPI-anchored SKS3 and CRK10 encoded by alternatively spliced transcriptional variants. **(A) **Alternative splicing of *SKS3*. **(B) **Alternative splicing of *CRK10*. ExD, extracellular domain; TM, transmembrane domain; KD, intracellular kinase domain. Exons are dark gray, introns are black lines, and untranslated transcribed regions are light gray.

In **Table 1**, 248 genes predicted to encode GPI-APs in 2003 have been listed. Some corrections have been made, as some of them could not be found in databases or turned out to encode non-coding RNA. However, according to more recent experimental data, genes not included in 2003 also turned out to encode GPI-APs, such as At1g09460, At2g30933, At2g03505, and At4g13600 (Simpson et al., 2009), *LORELEI* (Tsukamoto et al., 2010), and *XYP2* (Motose et al., 2004). Interestingly, due to recent achievements on alternative splicing, transcriptional variants of *SKS3* (Zhou, 2019a) and *CRK10* (Grojean and Downes, 2010) have been found to encode GPI-APs besides their ordinarily reported proteins (**Figure 2**). Alternative splicing largely enhanced the diversity of transcriptome and proteome, and more and more genes (up to 80% according to recent RNA-seq achievements) have been found to be alternatively spliced in *Arabidopsis*, which could greatly increase the abundance of GPI-APs (Wang et al., 2009; Filichkin et al., 2010; Severing et al., 2011; Reddy et al., 2013; Lee and Rio, 2015; Bush et al., 2017).

In addition, 163 GPI-APs were predicted in 2016, and those not included in **Table 1** are listed in **Table 2**. In this study, a large proportion of possible GPI-APs were discounted as typical GPI-APs in spite of being predicted to possess a GPI signal at the C terminus by various bioinformatics tools. Some of those discounted were transmembrane proteins, such as PIN3 and PIN4 and some receptor-like kinases (RLKs), and the other were cytoplasmic proteins without N-terminal secretory signal peptide, such as SNARE family proteins (listed at the end of **Table 2**).

## Functional Diversity of GPI-APs in *Arabidopsis*

GPI-APs listed in **Tables 1** and **2** are from diverse families, such as cell wall structure proteins, proteases, enzymes, receptor-like proteins (RLPs), lipid transfer proteins, and GPI-anchored peptides, which imply a functional diversity of GPI-APs: indeed, they were found functional in most processes, such as cell wall composition, cell wall component synthesis, cell polar expansion, stress responses, hormone signaling responses, pathogen responses, stomatal development, pollen tube elongation, and double fertilization in *Arabidopsis*.

Among these GPI-APs, the arabinogalactan protein (AGP) family, LORELEI family, COBRA family, and some RLPs, were better characterized. AGP family proteins are ubiquitous cell wall components anchoring on the plasma membrane throughout the Plant Kingdom and abundantly decorated at their Hyp residues by arabinogalactan polysaccharides, which make them be one of the most complex families of macromolecules in plants and play roles in various processes (Schultz et al., 2000; Ellis et al., 2010; Marzec et al., 2015; Showalter and Basu, 2016; Losada and Herrero, 2019; Palacio-Lopez et al., 2019). COBRA families were reported to be involved in various processes by regulating cell wall synthesis in plants (Hochholdinger et al., 2008; Cao et al., 2012; Niu et al., 2015; Niu et al., 2018). LORELEI family proteins associate with cell surface RLK, which is essential not only for ligand recognition but also for RLK transport (Capron et al., 2008; Duan et al., 2010; Tsukamoto et al., 2010; Meng et al., 2012; Yu et al., 2012; Li et al., 2015; Li et al., 2016; Liao et al., 2017; Stegmann et al., 2017; Feng et al., 2018; Guo et al., 2018; Yin et al., 2018).

## Involvement of GPI-APs in Signaling Transduction in *Arabidopsis*

**Figure 3:**
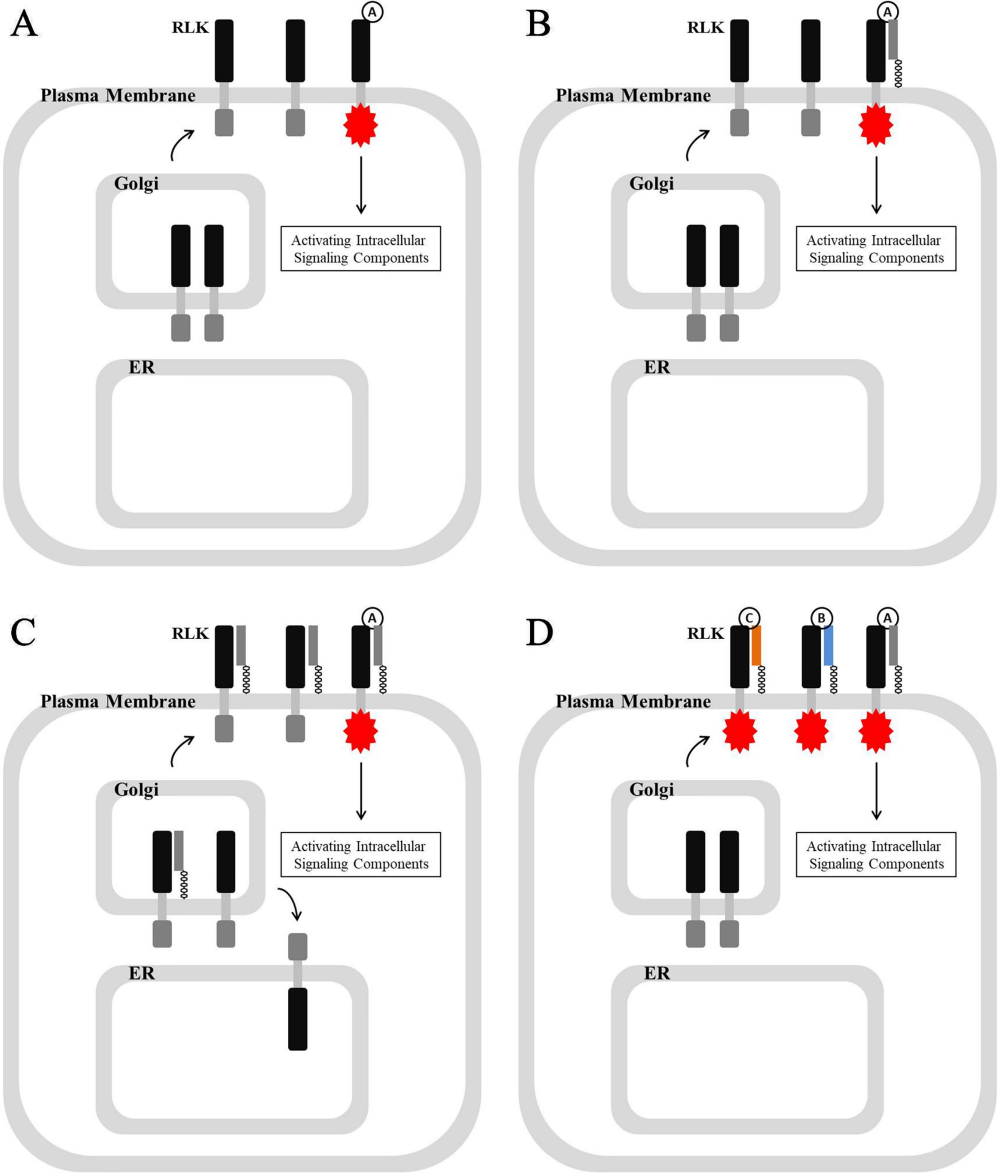
RLK-mediated signaling pathway and various associations between RLKs and GPI-APs. **(A)** Association with extracellular ligand activates and phosphorylates the intracellular kinase domain of RLK, which activates intracellular signaling components to regulate various processes. **(B)** GPI-AP is required for ligand recognition and its association with RLK. **(C)** GPI-AP is required not only for ligand recognition and its association with RLK but also for RLK localization by chaperoning its transport, and those un-chaperoned would reside in ER. **(D)** GPI-APs are required for ligand selection.

In *Arabidopsis*, hundreds of RLKs, which possess extracellular ligand recognition domains and intracellular kinase domains, control a wide range of processes, including development, disease resistance, hormone perception, and self-incompatibility (Shiu and Bleecker, 2001; Muschietti and Wengier, 2018; Wei and Li, 2018). Their association with extracellular ligands, including phytohormones, signaling polypeptides, and pathogen molecules, leads to the phosphorylation of the intracellular kinase domain, which consequently activate cytoplasmic signaling components and switch on response mechanisms (**Figure 3A**) (Pearce et al., 2001; Asai et al., 2002; Geldner and Robatzek, 2008; Murphy et al., 2012; Breiden and Simon, 2016; Yamaguchi et al., 2016; Chardin et al., 2017).

By summarizing the functional mechanism of those listed GPI-APs in **Tables 1** and **2**, a group of GPI-APs from various families was found to share a common mechanism of action involving RLK-related signal transduction (**Table 3**). The same mechanism has been reported in mammalian cells, for example, that GPI-anchored CD14 possessing leucine-rich repeats (LRR) region associates with not only Toll-like receptor TLR4 to perceive their polypeptide ligand lipopolysaccharide (LPS) leading them to activate mitogen-activated protein kinase (MAPK) cascades (Wright et al., 1990; Schumann, 1992; Zanoni et al., 2011; Li X. et al., 2015) but also TLR3 to perceive viral double-stranded RNA (dsRNA) leading them to activate (Vercammen et al., 2008). This common mechanism found in both animals and plants suggests that important and common roles are played by GPI-APs in signal transduction (**Figure 3B**).

**Table 3:** GPI-APs and their potential co-receptor RLKs and ligands.

	GPI-APs	Co-receptor RLKs	Ligands	Intracellular signaling components
Plants	SKU5	TMK1	ABP1	ROP GTPase (Shimomura, 2006; Xu et al., 2014)*
	LRE/LLGs	FERONIA	RALF1	RopGEFs-RAC/ROPs (Li et al., 2015; Li et al., 2016; Stegmann et al., 2017)
	FLA4	FEI1/FEI2	Unidentified	Unidentified (Seifert et al., 2014; Basu et al., 2016; Griffiths et al., 2016; Xue et al., 2017; Turupcu et al., 2018)
	ENODL14	FERONIA	Unidentified	Unidentified (Escobar-Restrepo et al., 2007; Hou et al., 2016)
	LRX5	FERONIA	RALF22/23	RopGEFs-RAC/ROPs (Zhao et al., 2018)
	TMM	ERf	EPFs	MAPK (Geisler et al., 1998; Geisler et al., 2000; Kobe and Kajava, 2001; Jakoby et al., 2006; Wang et al., 2008; Bhave et al., 2009; Abrash and Bergmann, 2010; Rasmussen et al., 2011; Lee et al., 2012; Yan et al., 2014; Lee et al., 2015; Meng et al., 2015; Bundy et al., 2016; Jorda et al., 2016; Lin et al., 2017)
	LYM1/LYM3	CERK1	Peptidoglycans	Unidentified (Willmann et al., 2011)
	LLG1	FLS2	flg22	Heterotrimeric G proteins (Liang et al., 2016; Shen et al., 2017)
Mammals	CD14	TLR4	LPS	MAPK (Wright et al., 1990; Schumann, 1992; Zanoni et al., 2011; Li et al., 2015)
	CD14	TLR3	Viral dsRNA	Lipid kinase PI3K (Vercammen et al., 2008)

*Both SKU5 and TMK1 were identified through their association with ABP1, but no direct association has been identified between TMK1 and SKU5.

## Association Between GPI-AP and RLK

Interestingly, the association between GPI-AP and RLK could be involved in not only ligand recognition but also RLK transport and subcellular localization. One of the best characterized GPI-APs, LORELEI, not only participates in ligand recognition by associating with FERONIA but also plays a crucial role in chaperoning the transport of FERONIA from the ER to the plasma membrane (Capron et al., 2008; Duan et al., 2010; Tsukamoto et al., 2010; Meng et al., 2012; Yu et al., 2012; Li et al., 2015; Li et al., 2016; Liao et al., 2017; Stegmann et al., 2017; Feng et al., 2018; Guo et al., 2018; Yin et al., 2018) (**Figure 3C**). This special chaperone and transport mechanism might be due to the GPI-APs becoming involved with lipid rafts, which determine distinct protein sorting and protein traffic (Eisenhaber et al., 1998; Legler et al., 2005; Miyagawa-Yamaguchi et al., 2015; Sezgin et al., 2017).

GPI-APs appear to be important not only for ligand recognition but also essential for ligand selection. For example, RLK FERONIA recognizes ligands RALF1 or RALF22/23 when associated with GPI-anchored LORELEI or LRX5, respectively (Li C et al., 2015; Li et al., 2016; Zhao et al., 2018). This potential GPI-AP-dependent selection mechanism could greatly enhance the ligand recognition abundance of RLK but could also mediate the cross-talk between various signaling perception and transduction (**Figure 3D**).

The associations between GPI-AP and RLK could be structure independent, such as SKU5-TMK1, LRE/LLGs-FERONIA, FLA4-FEI1/FEI2, ENODL14-FERONIA, and LRX5-FERONIA, or structure dependent, such as TMM and ERECTA both possessing LRR structure at the extracellular domain and LYM1/LYM3 and CERK1 both possessing LyM structure at extracellular domain in *Arabidopsis*. Interestingly, the same structure dependence is also present in one of the best characterized GPI-APs in mammalian cells, CD-14, and together with its partner receptor kinases TLR3 and TLR4 all possess an LRR structure.

The structure-dependent associations between GPI-APs and RLKs largely increased the curiosities of the group of GPI-anchored RLPs, which shared the same structures or sequence similarities with RLKs but lack intracellular kinase domains. They might recognize specific RLKs depending on sequence and structure similarities and form heterodimers with various RLKs in the ER or Golgi bodies and then chaperone them to specific plasma membrane regions through GPI-AP-driven lipid rafts. On arrival, they select and recognize ligands and activate the intracellular signaling components.

Whether the GPI-anchored RLKs encoded by transcriptional variants, such as GPI-CRK10 and its variant of CRK10, can form homodimers based on the same extracellular domains and play a role in RLK regulation, is a very interesting question.

## Conclusion and Perspectives

Previous genomic and proteomic assays that predicted and identified GPI-APs from *Arabidopsis* have been listed. Due to recent experimental data and knowledge of alternative splicing, more and more GPI-APs have been identified, suggesting that GPI-APs in *Arabidopsis* might be more abundant than we expected.

Previous studies on those listed GPI-APs from diverse families were discussed, and they were found to be involved in diverse biological processes, including cell wall composition, cell wall component synthesis, cell polar expansion, hormone signaling response, stress response, pathogen response, stomata development, pollen tube elongation, and double fertilization. Those reports demonstrated the functional diversity and indispensability of GPI-APs in *Arabidopsis*.

Among these reports, direct associations were found between various GPI-APs and their partner cell surface RLKs, demonstrating not only participation in their ligand recognition but also essential roles in RLK transport and localization. Localization might due to specific protein sorting and protein traffic driven by GPI-AP-related lipid rafts. Surprisingly, GPI-APs have also been shown to participate in ligand selection, which made one RLK and its downstream intracellular target activated by various ligands. Such protein cross-reactivity greatly enhanced the ligand recognition abundance of RLKs, which can also be considered as a common mechanism of cross-talk between various ligands or various signaling pathways.

In this review, the most predicted or identified GPI-APs in *Arabidopsis* were listed and discussed, and a common involvement of them in signing transduction was summarized. This involvement could be very helpful for understanding the ligand-RLK signaling transduction in plants, especially for understanding the polar localization of RLKs, and the crosstalk between various ligand-RLK signaling transduction. It would be interesting to identify more associations between various GPI-APs and RLKs and study their recognition and selection of ligands and downstream intracellular signaling components in *Arabidopsis*.

## Author Contributions

KZ wrote this manuscript.

## Conflict of Interest Statement

The authors declare that the research was conducted in the absence of any commercial or financial relationships that could be construed as a potential conflict of interest.
